# Supplementary material for: The R.O.A.D. to precision medicine
Source: NPJ Digit Med. 2024 Nov 3;7:307. doi: 10.1038/s41746-024-01291-6 (PMC11532393; doi:10.1038/s41746-024-01291-6)

Supplementary Material

Supplementary Figure 1 – Graphical representation of the changes in sensitivity and specificity with weight tuning using locally estimated scatterplot smoothing. Panels: A) the entire Polish validation cohort, B) the training cohort of the GIST genetic OPT, and C) the validation cohort of the GIST genetic OPT.

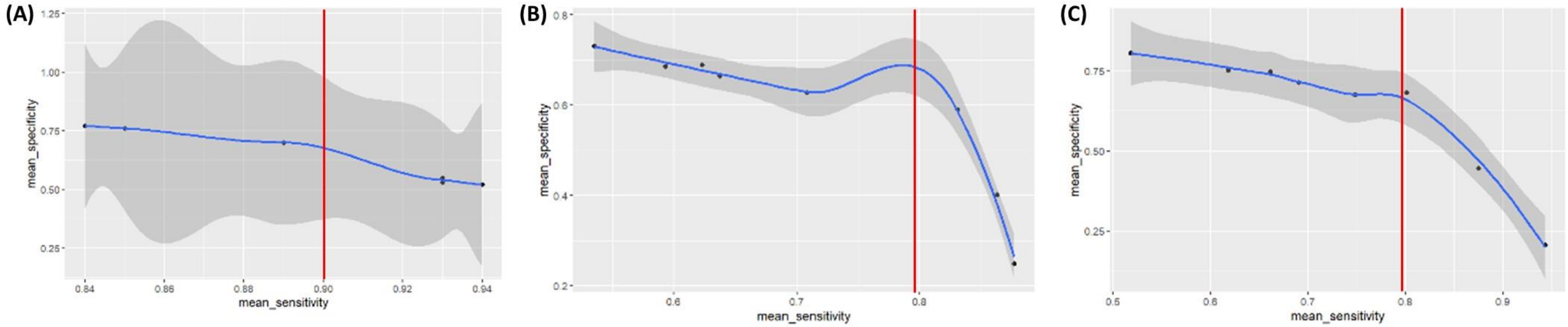

Supplement: Supplementary file 1 — Supplementary Figure 1 [file 41746_2024_1291_MOESM1_ESM.pdf]
